# Immunohistochemistry and Radiomic Features for Survival Prediction in Small Cell Lung Cancer

**DOI:** 10.3389/fonc.2020.01161

**Published:** 2020-08-12

**Authors:** Eleni Gkika, Matthias Benndorf, Benedict Oerther, Farid Mohammad, Susanne Beitinger, Sonja Adebahr, Montserrat Carles, Tanja Schimek-Jasch, Constantinos Zamboglou, Björn C. Frye, Fabian Bamberg, Cornelius F. Waller, Martin Werner, Anca L. Grosu, Ursula Nestle, Gian Kayser

**Affiliations:** ^1^Department of Radiation Oncology, Medical Center, University Hospital Freiburg, Freiburg, Germany; ^2^Department of Radiology Freiburg, University Medical Center Freiburg, Freiburg, Germany; ^3^Department of Neurology, Medical Center, University Hospital Freiburg, Freiburg, Germany; ^4^German Cancer Consortium (DKTK), Freiburg, Germany; ^5^German Cancer Research Center (DKFZ), Heidelberg, Germany; ^6^Department of Pneumology, Medical Center, University Hospital Freiburg, Freiburg, Germany; ^7^Faculty of Medicine, University of Freiburg, Freiburg, Germany; ^8^Department of Hematology, Oncology and Stem Cell Transplantation, University Medical Center Freiburg, Freiburg, Germany; ^9^Department of Pathology, Faculty of Medicine, Medical Center, Institute of Surgical Pathology, University Hospital Freiburg, Freiburg, Germany; ^10^Department of Radiation Oncology, Kliniken Maria Hilf, Mönchengladbach, Germany

**Keywords:** SCLC, immunohistochemistry, radiotherapy, radiomics, biomarkers, synaptophysin

## Abstract

**Background:** The aim of the study was to evaluate the role of different immunohistochemical and radiomics features in patients with small cell lung cancer (SCLC).

**Methods:** Consecutive patients with histologically proven SCLC with limited (*n* = 47, 48%) or extensive disease (*n* = 51, 52%) treated with radiotherapy and chemotherapy at our department were included in the analysis. The expression of different immunohistochemical markers from the initial tissue biopsy, such as CD56, CD44, chromogranin A, synaptophysin, TTF-1, GLUT-1, Hif-1 a, PD-1, and PD-L1, and MIB-1/KI-67 as well as LDH und NSE from the initial blood sample were evaluated. H-scores were additionally generated for CD44, Hif-1a, and GLUT-1. A total of 72 computer tomography (CT) radiomics texture features from a homogenous subgroup (*n* = 31) of patients were correlated with the immunohistochemistry, the survival (OS), and the progression-free survival (PFS).

**Results:** The median OS, calculated from diagnosis, was 21 months for patients with limited disease and 13 months for patients with extensive disease. The expression of synaptophysin correlated with a better OS (HR 0.546 95% CI 0.308–0.966, *p* = 0.03). The expression of TTF-1 (HR 0.286, 95% CI: 0.117–0.698, *p* = 0.006) and a lower GLUT-1 H-score (median = 50, HR: 0.511, 95% CI: 0.260–1.003, *p* = 0.05) correlated with a better PFS. Patients without chromogranin A expression had a higher risk for developing cerebral metastases (*p* = 0.02) and patients with PD 1 expression were at risk for developing metastases (*p* = 0.02). Our radiomics analysis did not reveal a single texture feature that correlated highly with OS or PFS. Correlation coefficients ranged between −0.48 and 0.39 for OS and between −0.46 and 0.38 for PFS.

**Conclusions:** The role of synaptophysin should be further evaluated as synaptophysin-negative patients might profit from treatment intensification. We report an, at most, moderate correlation of radiomics features with overall and progression free survival and no correlation with the expression of different immunohistochemical markers.

## Introduction

SCLC is an aggressive, high-grade neuroendocrine tumor associated with a short doubling time, a high growth fraction, and early development of widespread metastases, which contribute to the extremely poor prognosis of the disease (1–3). In an era of precision medicine, there is an increasing need for defining biomarkers that could assist in therapeutic decision making. Immunohistochemistry (IHC) is a widely available technique that can provide quickly and cost-efficiently clinically significant results in terms of diagnosis. Although some of the cytomorphologic features of small cell lung cancer (SCLC) are well-defined in the literature, little is known about their predictive or prognostic value.

Currently, synaptophysin, chromogranin A, and CD 56 are neuroendocrine markers routinely used for the diagnosis of SCLC (4, 5). However, 10–15% of the neuroendocrine markers have variable expressions or lack expression (6–8). Pulmonary SCLC show thyroid transcription factor-1 (TTF-1) expression in more than 90% (9, 10) similar to CD56, which stains ~90–100% of the cases. In addition, 25–37% of SCLC cells show a positive staining for CD 44 (11, 12) and about 78–92% of the patients show a glucose transporter 1 (GLUT-1) expression (13–15). Tumors with a rapid doubling time typically have a high mitotic rate and a proliferation index of 70–90% (5, 8, 16). The role of hypoxia-inducible factor 1a (HIF-1a) in SCLC is not well-defined. Wan et al. suggest that HIF-1a may enhance the angiogenic potential of SCLC by regulating some angiogenic genes, such as the vascular endothelial growth factor VEGF-A (17), but literature is limited. Programmed cell death ligand 1 (PD-L1) is a predictive biomarker for immunotherapy in several solid tumors, but its role in the treatment of SCLC is not well-defined (18–22).

It has been suggested that the intra-tumoral heterogeneity within solid cancers could be captured with the use of medical imaging, which might enhance our understanding of the tumor biology (23, 24). The use of radiomics might provide additional information to the immunohistochemistry concerning the phenotype of the tumor. In this analysis, we evaluate the potential role of different CT radiomic features and immunohistochemical markers, such as CD56, chromogranin A, synaptophysin, TTF1, Ki 67, CD44, HIF-1a, GLUT-1, PD 1, and PD-L1, in patients with SCLC.

## Methods and Materials

### Patient Characteristics

The study was approved by the local ethics committee. Consecutive routine patients with histologically proven SCLC receiving radiation therapy and chemotherapy at our department were included in this study. Computer tomographies (CTs) of the thorax and abdomen, bone scans, as well as magnetic resonance imaging (MRI) of the brain were performed during the initial routine workup. Tumors were staged and assessed according to the Union Internationale Contre le Cancer (UICC) seventh edition.

Patients were routinely evaluated during treatment. Complete blood tests, including blood count, and biochemical analysis, such as liver and renal function tests, lactate dehydrogenase (LDH, [U/l], Supplementary Table 1) and tumor markers, such as neuron-specific enolase (NSE, [μg/l], Supplementary Table 1) were assessed routinely. Follow-up visits were scheduled every 3 months, including physical examination and CTs. Tumor response was assessed according to the Response Evaluation Criteria In Solid Tumors (RECIST) criteria (25).

### Treatment Characteristics

Chemotherapy typically consists of four to six cycles of cisplatin-based doublets (or carboplatin in the presence of extensive disease or comorbidities) in combination with etoposide administered every 21 days. In the absence of metastases, chemoradiation (50.4 to 66 Gy in 1.8–2 Gy daily fractions) was delivered concurrently with the second cycle of chemotherapy. In case of symptom palliation, 30–36 Gy in 3-Gy daily fractions were delivered. Patients without cerebral metastases with or without distant metastases who responded after initial treatment received prophylactic cranial irradiation (PCI) up to a total dose of 30 Gy, according to national guidelines.

### Immunohistochemistry

Formalin-fixed paraffin-embedded tissue samples were cut into 3-μm slices and mounted on positively charged glass slides. Immunohistochemistry was performed using a Dako Autostainer Plus-Link (Dako, Glostrup, Denmark). Details of the antibodies and staining protocols used are listed in Supplementary Table 2.

For all antibodies, specific staining patterns [i.e., nuclear for KI67, TTF-1, membranous for CD56, GLUT1, PD-L1 (tumor cells, macrophages, stroma) and cytoplasmic for CD 44, Chromogranin, PD-1] were evaluated. Any intensity was recorded. TTF-1, CD56, Chromogranin A, and Synaptophysin were classified as positive if the majority of tumor cells showed specific staining reactivity.

For CD44, GLUT-1, and HIF-1a, H-scores were calculated according to the following formula (26, 27):

[1 × (% cells 1+) + 2 × (% cells 2+) + 3 × (% cells 3+)].

All viable tumor cells within the biopsy were taken into account when applying the semi-quantitative scoring system.

### Radiomics Subgroup Analysis

We performed a radiomics analysis in a homogenous patient subgroup, concerning the CT scans, performed either with a Siemens Sensation 16 or a Siemens Sensation 64 scanner (*n* = 31, *n* = 13 at Siemens Sensation 16, *n* = 18 at Siemens Sensation 64). All patients received intravenous contrast agent (mean: 82 ml, range: 60–100 ml, similar in both scanners; *p* = 0.93, two-sided *t* test). Images were analyzed in 3-mm lung kernel reconstructed slices. All images had a matrix of 512 × 512 voxels. Comparison (two sided *t* test) of mean HU (and standard deviation, SD) of segmented tumors between the two scanners to identify a possible systematic bias of gray values analyzed resulted in a *p*-value of 0.45 (0.07 SD).

**Figure 1:**
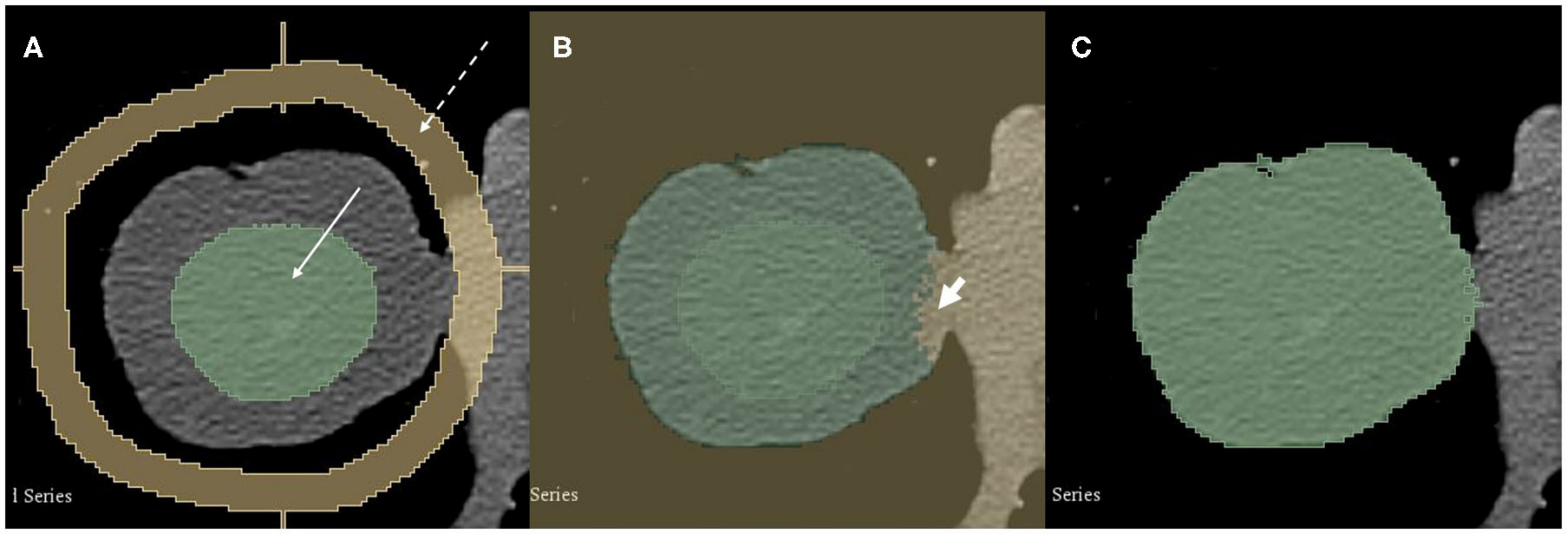
A 74-year-old male patient with pT2 pN0 cM0 R0 G3 SCLC in the middle lobe. Semi-automated segmentation process. **(A)** A radiologist manually set seeds in the tumor (green, solid arrow) and the surrounding tissue (yellow, dashed arrow). **(B)** The grow-cut algorithm is applied and automatically segments the tumor. Note small error on the medial side (short arrow). Such errors are corrected manually. **(C)** Final tumor segmentation.

The primary tumor was predefined by a radiation oncologist, and three-dimensional, semi-automated segmentation was performed by a radiologist in the axial images. Segmentation was performed in a 3-D slicer (28), using the grow-cut algorithm (29). This algorithm has been demonstrated to generate more robust segmentation results than manual definition of tumor volume for NSCLC cases (29). A radiologist manually sets seeds within the tumor and in the surrounding different tissues, e.g., mediastinum, lung, and chest wall. The algorithm then grows regions of interest that fit best to the set seeds according to voxel gray value (Hounsfield units). Results were inspected and, in case of obvious flaws, were manually corrected by the radiologist. An example of the semiautomated tumor segmentation is provided in Figure 1.

We employed the radiomics pipeline implemented in the PyRadiomics package for Python to derive texture features (30). A total of 72 texture features was obtained for each tumor (all features from the implemented classes gray level co-occurrence matrix, gray level run length matrix, first order histogram features, and shape features were derived). We calculated correlation coefficients (Pearson correlation and Spearman correlation to account for possible non-linear associations) for the single texture features with overall survival and progression-free survival.

### Statistical Analysis

Overall survival (OS) was calculated as time from pathological confirmation until death from any cause with censoring at the date last seen alive. Progression-free survival (PFS) was calculated as time from start of treatment until death or documentation of progression. OS and PFS were estimated by the Kaplan-Meier method. The Cox proportional hazards regression model was used for further analyses of possible prognostic factors for OS and PFS. All immunohistochemical markers were regarded as categorical variables, H scores, MIB-1, NSE, and LDH were regarded as continuous variables and were reported as median with the corresponding range (minimum and maximum). Univariate and multivariate analysis was performed with the Cox proportional hazards model. For multivariate analysis, we included in the model only the parameters that were statistically significant in the univariate analysis. Results are reported as hazard ratios (HR), 95% confidence intervals (CI), and *p*-values.

Concerning radiomics analysis, the Pearson and Spearman correlation coefficients were used for correlation of single texture features with OS and PFS to account for possible non-linear associations. Due to the small sample size and general recommendation to incorporate around 20 cases per explanatory variable in multivariate regression models (31), we build bivariate Cox proportional-hazard models with the two radiomics features correlated most with OS (model OS_radiomics_) and PFS (model PFS_radiomics_). We required features to be moderately autocorrelated at most. Furthermore, we build bivariate models with the radiomics feature that exhibits strongest correlation with OS/PFS and NSE and LDH serum level as additional variables (models OS_radiomics−NSE_, OS_radiomics−LDH_, PFS_radiomics−NSE_, and PFS_radiomics−LDH_. Univariate models are generated for all models for comparison.

The statistical significance level was set at ≤ 0.05. All *p*-values were two-sided. Statistical analysis was performed using SPSS version 23 (SPSS, Chicago IL).

## Results

### Patient and Treatment Characteristics

Consecutive patients (*n* = 101) with evaluable pathological specimens (*n* = 98) in our pathology department treated in the radiation oncology department between 2004 and 2014 were included in the analysis. A disseminated disease was present at diagnosis in 51 (52%) patients, and 47 (48%) of the patients had no metastatic disease. Patient characteristics are shown in Table 1.

**Table 1:** Patient and treatment characteristics.

**Variable**	**Nr (%) or median (range)**
Age (years)	64 (42-84)
Gender	
Male	63 (64%)
Female	35 (36%)
TNM	
T	
T1	7 (7%)
T2	15 (15%)
T3	23 (24%)
T4	53 (54%)
N	
N0	10 (10%)
N1	6 (6%)
N2	31 (32%)
N3	51 (52%)
M	
M0	47 (48%)
M1	51 (52%)
Stadium	
I	4 (4%)
II	6 (6%)
III	37 (38%)
IV	51 (52 %)
ECOG	
1	60 (61%)
2	35 (36%)
n.s	3 (3%)
NSE [μg/l]	42.2 (10.8–597.0)
LDH [U/l]	258 (146–973)

*ECOG, Eastern Cooperative Oncology Group; NSE, neuron-specific enolase; LDH, lactate dehydrogenase*.

A total of 70 patients received a thoracic radiotherapy, of which 55 (56%) patients (M0 or M1a) were treated in curative intent with a median dose of 50.4 Gy and 15 patients for palliation with a median dose of 30 Gy. A total of 50 patients with good response after initial treatment, of which 37 had no signs of metastases and 13 with extracranial metastatic spread at diagnosis, received a prophylactic cranial irradiation and 31 patients a palliative whole brain irradiation due to cerebral metastases. Fifty-five (56%) patients received Carboplatin/Etoposide, and 30 (31%) patients Cisplatin/Etoposide. One patient did not receive any chemotherapy due to comorbidities, and 11 patients received different platinum-doublets in combination with topotecan (2%) or paclitaxel and etoposide (2%) or pemetrexed (3%) or analog to the EPICO protocol (epirubicin/cyclophosphamid/vincristin, 4%). One patient received topotecan alone.

### Overall Survival and Progression-Free Survival

The median follow-up was 73 months for patients alive. The median overall survival for the whole group was 15 months (95% CI: 12–18). The median survival of the patients without metastases was 21 (95% CI: 16–28) months and for patients with distant metastases 13 (95% CI: 11–15) months, respectively. The OS at 2 and 5 years was 33 and 13% for the patients without metastases and 14 and 0% for patients with metastatic disease, respectively. The median progression-free survival was 7 months.

Patients with higher LDH (HR 1.003, 95% CI 1.001–1.004, *p* < 0.0001) and NSE (HR 1.004, 95% CI 1.002–1.006, *p* = 0.001) concentrations had worse overall survival.

Patients treated with cisplatin doublets, compared to carboplatin, had better survival (HR 0.613, 95% CI 0.389–0.966, *p* = 0.035) probably due to selection bias as patients with comorbidities received carboplatin rather than cisplatin. Similarly, patients who received thoracic radiation (HR 0.488, 95% CI 0.307–0.776, *p* = 0.002) had better survival. Furthermore, patients who received a PCI had a prolonged survival (HR: 0.446, 95% CI 0.290–0.686, *p* < 0.0001), which was also significant in multivariate analysis. This result remained significant for the patients without metastases at baseline (HR 0.202 95% CI: 0.087–0.472, *p* < 0.0001) but not for the patients with disseminated disease (HR 0.846, 95% CI 0.446–1.607, *p* = 0.51). Patients without metastases who received PCI had a median OS of 23 (95% CI: 20–26) months vs. 10 (95% CI: 9–12) months for patients without PCI. PCI was also associated with a longer PFS (HR: 0.156, 95% CI: 0.055–0.442, *p* < 0.0001). Eleven patients who were treated with PCI developed cerebral metastases.

### Immunohistochemical Markers

**Figure 2:**
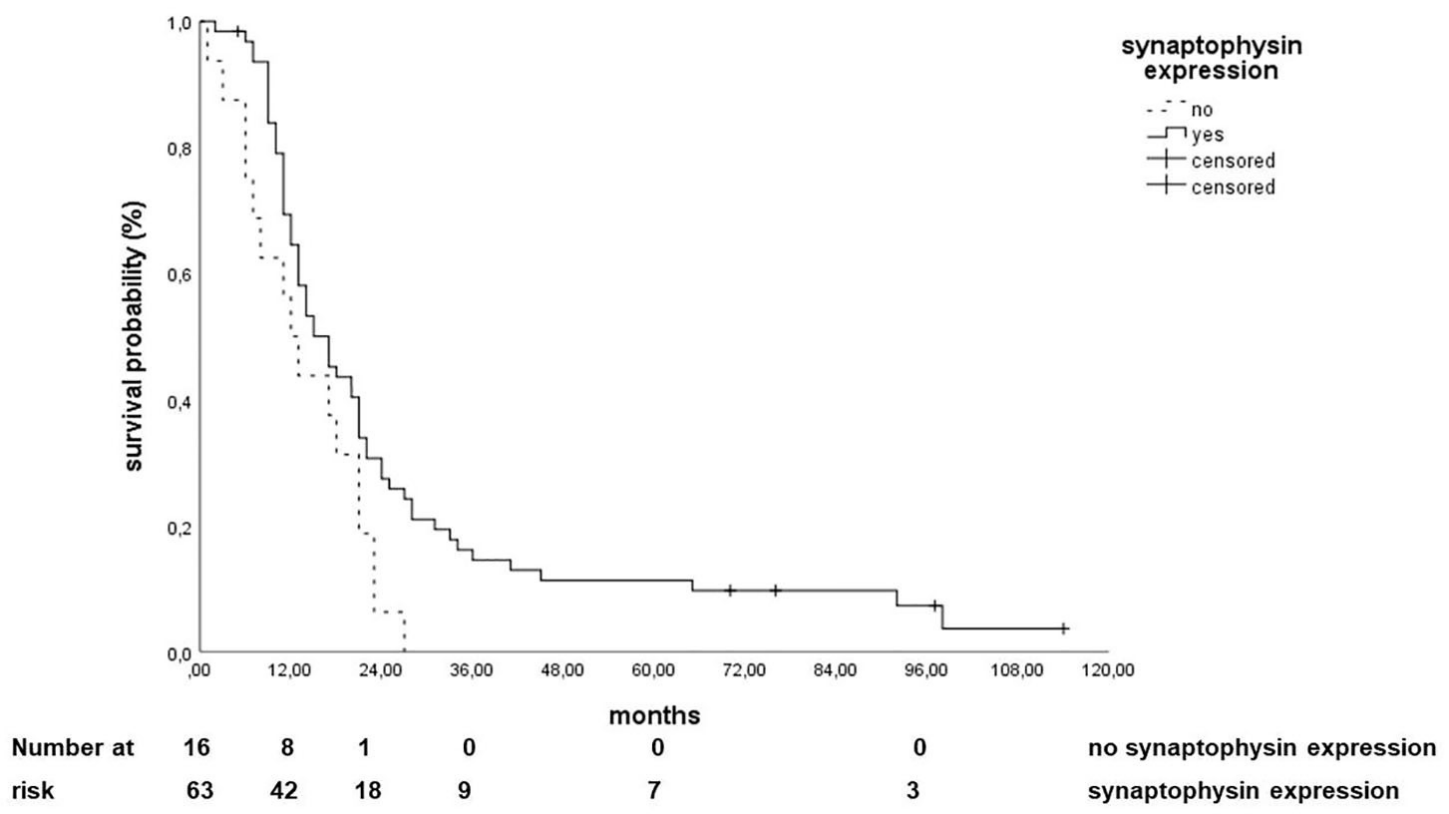
Correlation of synaptophysin expression with overall survival.

A total of 98% of the biopsies were positive for CD56, 63% for chromogranin A, and 80% for synaptophysin. About 90% were positive for TTF-1, 100% for CD44 and Hif-1a, and 84 for GLUT-1 and 72% for PD-1. The different immunohistochemical markers and H scores as well as their distribution between metastatic and non-metastatic disease are shown in Tables 2A,B. Only the expression of synaptophysin correlated with a better OS (HR 0.546 95% CI 0.308–0.966, *p* = 0.039). Patients without an expression of synaptophysin had a median OS of 12 (95% CI: 8.1–15.9) months, whereas patients with a synaptophysin expression had a median OS of 17 months (95% CI: 13–21) (Figure 2). None of the other markers or the H scores correlated with a better OS (Table 3). These results were not statistically significant on multivariate analysis (Table 4).

**Table 2A:** Immunhistochemical markers.

	**All patients**		**M0 patients**		**M1 patients**		**p = 0**
**Marker**	**Nr of patients**	**Nr positiv(%)**	**Nr of patients**	**Nr positiv(%)**	**Nr of patients**	**Nr positiv(%)**	
CD56	89	87 (98%)	45	45 (100%)	44	42 (96%)	0.99
Chromogranin A	57	36 (63%)	27	19 (70%)	30	17 (57%)	0.28
Synaptophysin	79	63 (80%)	37	33 (89%)	42	30 (71%)	0.06
TTF1	71	64 (90%)	41	37(90%)	30	27 (90%)	0.97
Hif-1-alpha	44	44 (100)	29	29 (100%)	15	15 (100%)	n.a.
CD 44	44	44 (100)	29	29 (100%)	15	15 (100%)	n.a.
GLUT-1	36	43 (84%)	28	25 (89%)	15	11 (73%)	0.19
PD-1,	18	13 (72%)	12	11 (92%)	6	2 (33%)	0.02
PD-L1 tumor	19	2 (10%)	13	2 (15%)	6	0 (0%)	0.80
PD-L1 stroma	19	6 (32%)	13	5 (38%)	6	1 (17%)	0.36
PD-L1 macrophages	19	12 (63%)	13	10 (76%)	6	2 (33%)	0.08
MIB-1	34	n.a.	12	n.a.	22	n.a.	0.86

*n.a., not applicable*.

**Table 2B:** H-scores and MIB-1.

**Biomarker H-Scores**	**All patients**	**M0 patients**	**M1 patients**
	**Median (range)**	**Median (range)**	**Median (range)**
Hif-1a	190 (20-300)	190 (20-300)	210 (110-270)
CD 44	45 (10-260)	40 (10-240)	50 (20-260)
GLUT-1	50 (0-160)	50 (0-160)	50 (0-140)
MIB-1	70 (30-100)	80 (30-95)	70 (35-100)

**Table 3:** Univariate analysis of different immunohistochemical markers concerning overall survival.

**Immunhistochemical marker**	**Hazard Ratio (95% CI)**	***p***
CD56	0.295 (0.071–1.226)	0.09
Chromogranin-A	0.749 (0.430–1.303)	0.31
Synaptophysin	0.546 (0.308–0.966)	0.03
TTF-1	0.546 (0.247–1.211)	0.14
Hif1a H score	1.000 (0.993–1.006)	0.95
GLUT-1 H score	1.006 (0.998–1.013)	0.13
PD-L1 expression	0.933 (0.829–1.050)	0.25
MIB-1	1.001 (0.982–1.021)	0.90

**Table 4:** Multivariate analysis of different parameters concerning overall survival.

**Variable**	**Hazard Ratio (95% CI)**	***p***
LDH^*^	1.001 (0.999–1.004)	0.374
NSE^*^	1.002 (0.999–1.005)	0.273
Synaptophysin	0.914 (0.479–1.743)	0.785
PCI	0.477 (0.265–0.860)	0.014
M1	0.540 (0.258–1.129)	0.102
Cisplatin based doublets	0.555 (0.307–1.002)	0.051
Thoracic radiotherapy	0.532 (0.276–1.025)	0.059

* *Continuous variable*.

*PCI, prophylactic cranial irradiation; NSE, neuron specific enolase; LDH, lactate dehydrogenase*.

**Figure 3:**
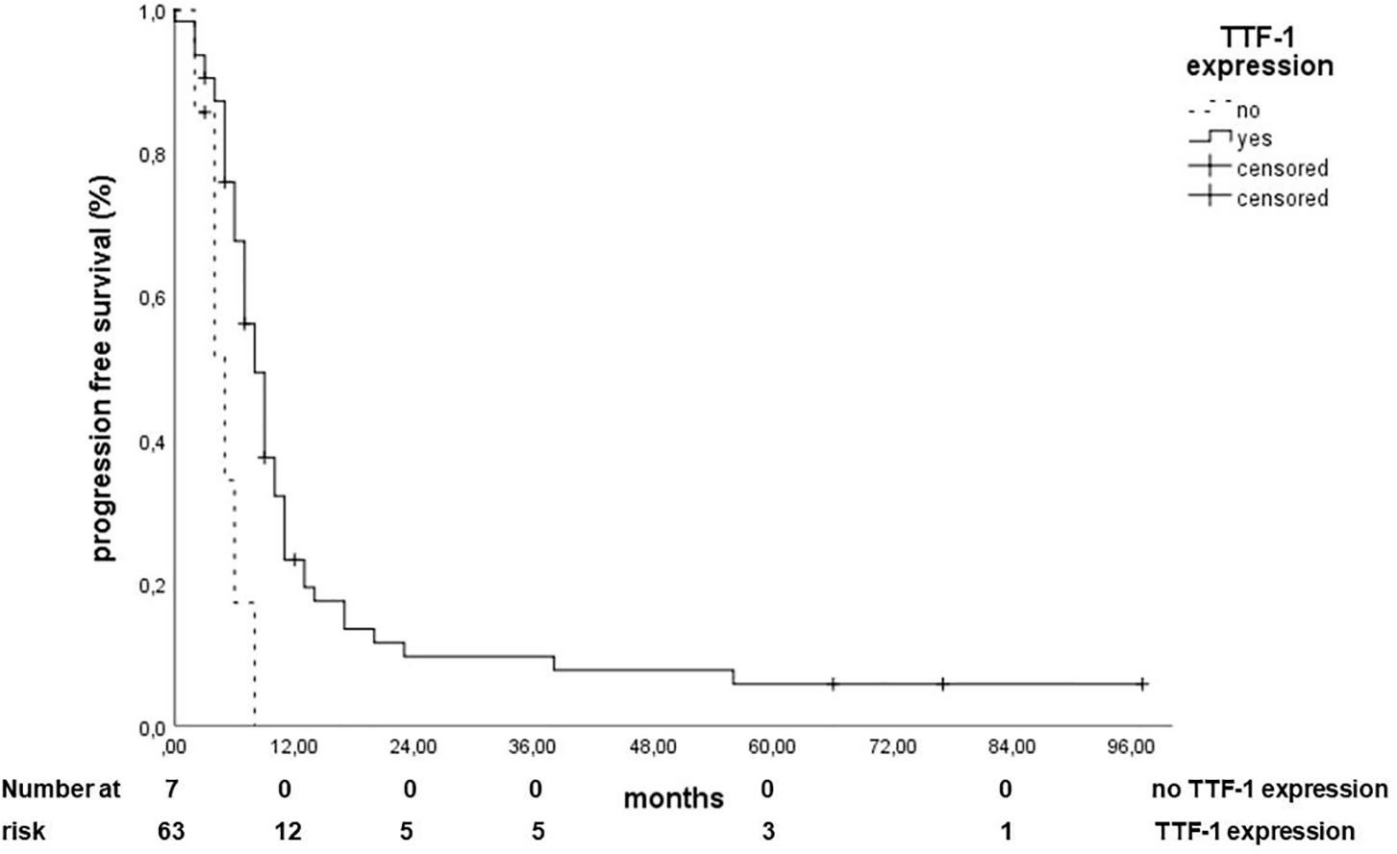
Correlation of TTF 1 expression with progression-free survival.

**Figure 4:**
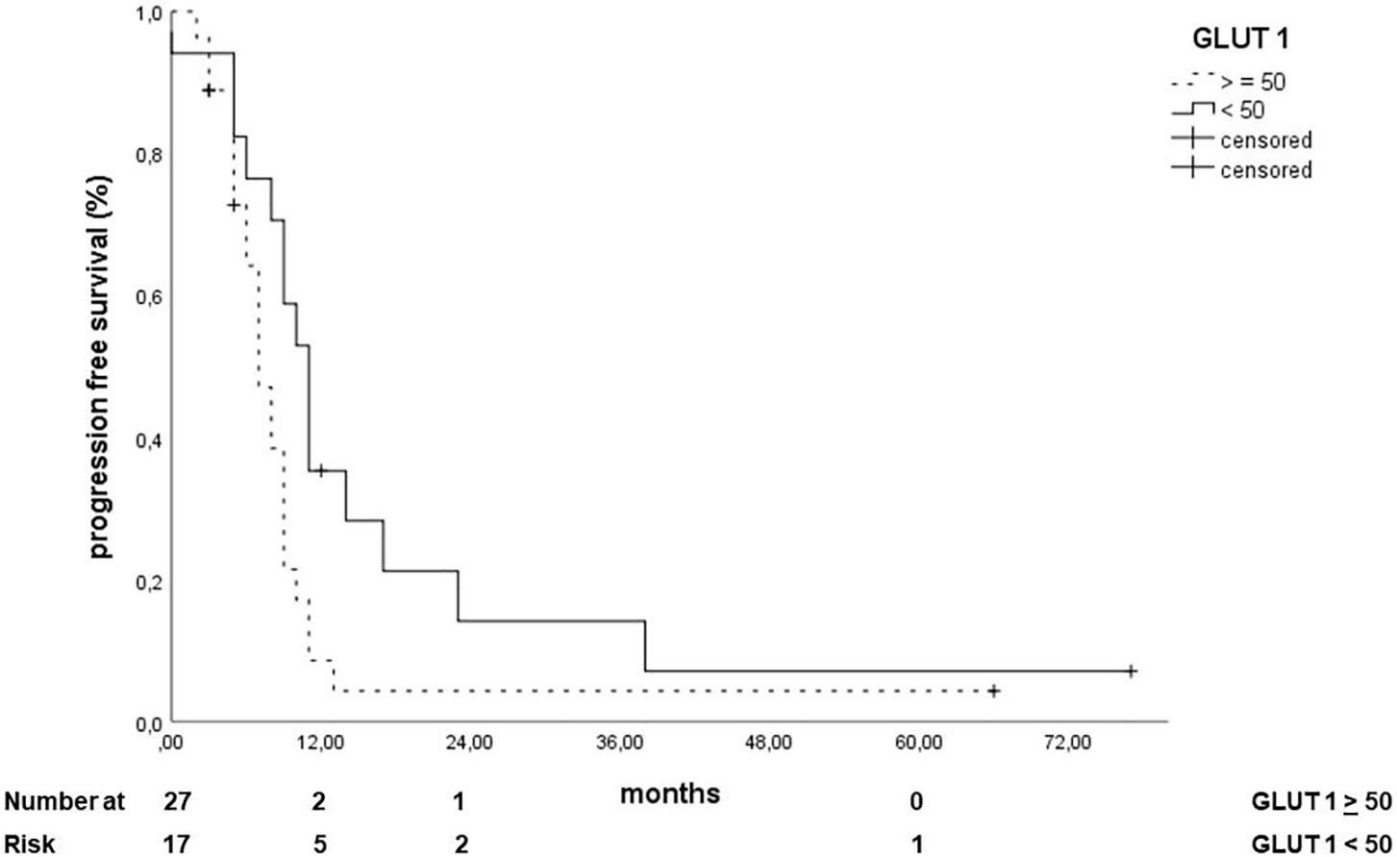
Correlation of GLUT 1 expression (over the median) with progression-free survival.

Concerning the PFS, only the expression of TTF-1 (HR 0.286, 95% CI: 0.117–0.698, *p* = 0.006, Figure 3) and a GLUT-1 H score lower than the median (median = 50, HR: 0.511, 95% CI: 0.260–1.003, *p* = 0.05, Figure 4) correlated with a better PFS.

None of the immunohistochemical markers except for PD 1 (HR 22.000, 95% CI 1.540–314.292, Table 2A) correlated with the presence of metastases at diagnosis, and the lack of chromogranin A expression correlated with the presence of cerebral metastases (HR 0.250, 95% CI: 0.079–0.787, *p* = 0.02).

### Radiomic Features

Our radiomics analysis did not reveal a single texture feature that was highly correlated with overall or progression-free survival. Results are provided in Supplementary Table 3.

**Figure 5:**
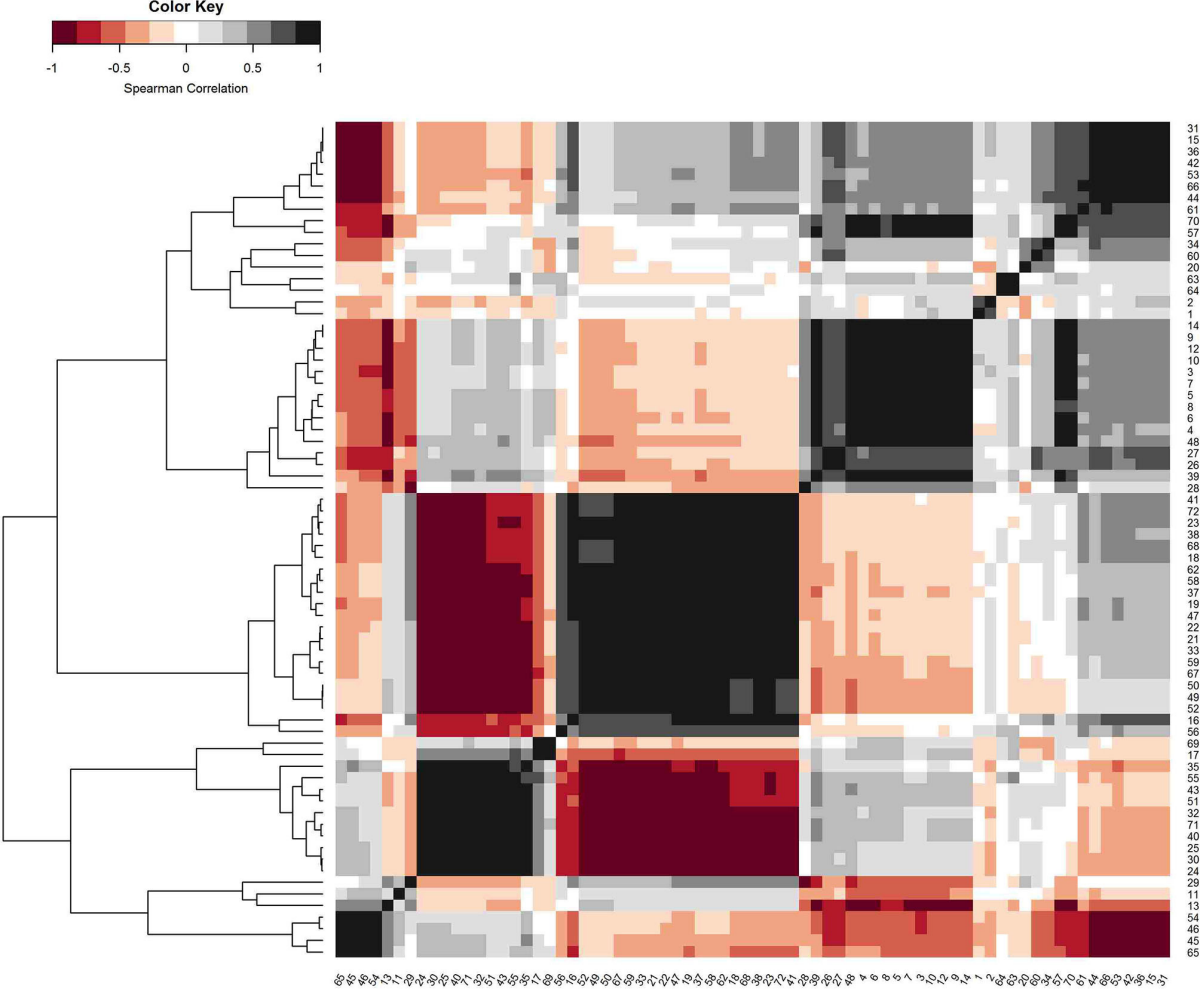
Correlation matrix (Spearman correlation) of the derived radiomics features. Features were ordered with a hierarchical agglomerative clustering algorithm to visualize clusters of collinearity to allow for variable selection.

Correlation coefficients ranged between −0.48 and 0.39 for overall survival and between −0.46 and 0.38 for progression-free survival (Spearman correlation coefficients in each case). Figure 5 shows the correlation matrix of the derived radiomics features; the numbers refer to the respective features in Supplementary Table 1.

Highest correlation coefficients for OS were obtained for features original_glcm_Imc1 (*r* = −0.48), original_glcm_Imc2 (*r* = 0.40, high correlation with original_glcm_Imc1 with *r* = −0.95, compare with Figure 5) and original_shape_Maximum2DDiameterColumn (*r* = −0.38, correlation with original_glcm_Imc1 *r* = 0.41; these two features were taken for bivariate model building; OS_radiomics_). The same features were identified for the correlation with progression-free survival, resulting in model PFS_radiomics_. Serum NSE and LDH level was added to the feature with strongest correlation (original_glcm_Imc1), resulting in bivariate models OS_radiomics−NSE_, OS_radiomics−LDH_, PFS_radiomics−NSE_, and PFS_radiomics−LDH_.

Results of the Cox proportional hazard models are provided in Tables 4 and 5 for overall survival as an outcome variable. Radiomics features were not able to model survival (both overall and progression free). Hazard ratio was significantly >1 for overall survival for NSE and LDH in univariate models; for PFS, *p*-values were 0.10 and 0.11 in univariate models, respectively.

**Table 5:** Cox-proportional hazard models for radiomics features and radiomics features and serum markers, overall survival as outcome variable.

**Feature/Model**	**Hazard Ratio (95% CI) OS**	***p***
original_glcm_Imc1	142,301 (2.141e-07–9.457e+16)	0.393
original_glcm_Imc2	0.1629 (0.005–5.219)	0.305
original_shape_Maximum2DDiameterColumn	1.01 (0.997–1.023)	0.141
NSE	1.004 (1.0–1.007)	**0.038**
LDH	1.003 (1.0–1.006)	**0.029**
OS_radiomics_		
original_glcm_Imc1	7518.6 (2.666e-09–2.120e+16)	0.542
and		
original_shape_Maximum2DDiameterColumn	1.009 (9.954e-01–1.023)	0.191
OS_radiomics__+__NSE_		
original_glcm_Imc1	1.320e+05 (3.801e-08–4.585e+17)	0.424
and		
NSE	1.004 (1.0–1.008)	**0.035**
OS_radiomics__+__LDH_		
original_glcm_Imc1	1.449e+06 (6.95e-08–3.020e+19)	0.365
and		
LDH	1.003 (1.0–1.006)	**0.026**

*NSE: neuron specific enolase, LDH: lactate dehydrogenase, OS: overall survival. Bold values indicate statistically significant*.

An unpaired Wilcoxon signed rank test was performed to analyze whether the three identified radiomics features were associated with positive or negative synaptophysin / chromogranin A staining, which was negative for both tests (*p* > 0.05).

## Discussion

In the era of personalized medicine, the integration of molecular markers with radiomics features and clinical information to develop novel prognostic biomarkers for treatment response assessment has been the focus of several studies (32–39). Especially in non-small cell lung cancer (NSCLC), the concept of using radiomic features in combination with genomic or proteomic information aiming to enhance our understanding of the tumor phenotype has been reported in several studies (32, 40–42). Radiomics features extracted from CT were related to tumor metabolism, PET tumor stage, and histopathology in NSCLC (43, 44), but little is known about their role in SCLC. Similarly, the prognostic and predictive value of different immunohistochemical markers in the treatment response, the presence of metastases, or survival of SCLCs has not been widely investigated. Both IHC and CT have a number of advantages, including being widely available, technically less challenging, and cost-efficient. Thus, the information derived from both diagnostic tools could enhance our understanding of this aggressive and insufficiently investigated tumor entity.

In our analysis, the lack of synaptophysin expression correlated with a worse OS, contrary to what is reported for NSCLCs (45, 46), whereas lack of chromogranin A correlated with the presence of cerebral metastases at diagnosis. Both neuroendocrine markers play a significant role in the diagnosis, but little is known about the prognosis of SCLC who lack expression of neuroendocrine markers. Sloman et al. (12) investigated the role of several immunohistochemical markers, including synaptophysin and CD 44 in a small sample of 28 patients, and could not find a significant correlation concerning survival, whereas in an analysis by Drivsholm et al. (47), the expression of chromogranin A in plasma correlated with a worse OS and the stage of disease. Recent studies have shown that SCLC has four different molecular subtypes with a different neuroendocrine character each identified by their key transcriptional regulator (3, 48). A subset of small cell lung carcinomas shows loss of the typical neuroendocrine markers. Some of these phenotypes were found to be associated with poor prognosis and chemoresistance although, for other phenotypes, the clinical outcomes have not yet been defined (48, 49). A better understanding of the critical signaling pathways operant in particular SCLC subtypes may define vulnerabilities and therapeutic targets (48, 50).

The expression of TTF-1 correlated in our analysis with a better progression-free survival. Similar were the results in an analysis by Misch et al. (51) reporting that there was a correlation with the disease control rate (DCR) in patients with metastatic disease (stage IV). There was a significantly (*p* = 0.006) improved response to the treatment in the group of patients with TTF-1-expression (DCR 86 vs. 56%). They concluded that TTF-1 may serve as a predictor of response to first line chemotherapy.

The proliferation index measured by Ki 67/MIB 1 plays a major role in several malignancies (52–54), but there was no correlation found in our analysis with the OS or with the PFS. Although the Ki-67 protein is well-characterized on the molecular level and extensively used as a proliferation marker, the functional significance still remains unclear. There are indications, however, that a Ki-67 protein expression is an absolute requirement for progression through the cell-division cycle (55). Some studies (56–58) did not find a correlation with response after chemoradiation, similar to our results, but other studies show a correlation of the proliferation rate with the prognosis (59, 60).

The uptake of GLUT-1 has been found to be increased in several cancer types (13, 61) and is associated with a poorer survival outcome in NSCLC (62). Abnormal expression of GLUT-1 was significantly associated with poor differentiated tumors, positive lymph node metastasis, and larger tumor size, which suggests that overexpression of GLUT1 is linked with enhanced invasive potential, proliferative activity, and decreased patient survival (61). In a study by Ozbudak et al. (14) GLUT-1 was expressed in approximately half of the pulmonary neuroendocrine carcinomas and showed a strong correlation with neuroendocrine differentiation/grade. In our analysis a GLUT-1 H score lower than the median of 50 correlated with a better PFS, but no other association could be found for OS.

The activation of HIF-1a is generally more pronounced in aggressive tumors and can be an independent predictor of poor prognosis in certain types of cancer (17, 63). In our analysis, all tumor samples tested were HIF-1a positive. Similarly, 98% of the biopsies were positive for CD56. This high expression of CD56 in patients with SCLC seemed promising for the use of anti CD56 antibodies for the treatment of CD56 positive tumors, such as Lorvotuzumab mertasine (64). Furthermore, preclinical data indicate potent antitumor effects of CD56 chimeric antigen receptor (CAR) T-cells in SCLC (65–67).

The use of immunotherapies in the metastatic setting shows an improvement in OS and PFS in several studies (21, 22), but concerning the role of PD-L1 expresion, data are limited as a number of clinical trials with PD-1, PD-L1, or CTLA-4 inhibitors are still ongoing. Initial data from the CheckMate 032 study suggests no correlation between PD-L1 expression and clinical benefit (18). However, in the Keynote-028 and Keynote-158 studies of pembrolizumab (anti-PD1), PD-L1 expression, especially combined expression on tumor and immune stromal cells, was associated with improved response to pembrolizumab (35.7% vs. 6%) (19, 20). According to Schultheis et al. (68) the PD-1/PD-L1 pathway seems activated in a fraction of SCLCs. In their study, the carcinoma cells were negative in all cases; PD-L1 was expressed in tumor-infiltrating macrophages and was correlated with tumor-infiltrating lymphocytes. In another study, however, Ishii et al. (69) reported that the expression of PD-L1 in tumor cells as defined by >5% of positive cells were observed in 71.6% (73 of 102) SCLC cases. In our analysis, except for a correlation between PD 1 and the presence of metastases, we could not find any correlation between the expression of PD-1/PD-L1 and the OS, but results are not easy to interpret due to the very small sample size.

On the other hand, human cancers exhibit strong phenotypic differences that can be visualized non-invasively by medical imaging (70, 71). In our explorative radiomics analysis, single radiomics features did correlate moderately with OS and PFS. The small sample size limits the conclusions to be drawn from our findings. However, we decided to only include patients with homogenous CT examinations into the radiomics analysis since image acquisition parameters affect radiomics signatures to a considerable extent (72). In the developed Cox proportional hazard models, radiomics features were not able to significantly model survival while retained conventional serum markers NSE and LDH in the models behaved as expected.

The present study has several limitations, such as its retrospective and single institutional nature, the small sample size as well as a selection bias in the treatments delivered.

In conclusion, a high expression of the neuroendocrine marker synaptophysin correlated significantly with a better survival in patients with SCLC, whereas the expression of TTF-1 and a lower GLUT-1 H-score correlated with a better PFS. These results should be further evaluated as synaptophysin-negative tumors might have a poor prognosis and TTF1 negative tumors might be more resistant to current therapies. This might be due to different SCLC-phenotypes, who might profit from treatment intensification. These different markers were not associated with any radiomics features. Furthermore, we did not find a high correlation between single radiomic features with overall survival or progression-free survival as radiomics features were not able to model survival in the analyzed patient subset.

## Data Availability Statement

The datasets generated for this study are available on request to the corresponding author.

## Ethics Statement

The studies involving human participants were reviewed and approved by Ethic Committee University of Freiburg, Germany. The patients/participants provided their written informed consent to participate in this study.

## Author Contributions

EG, MB, BO, FM, SB, UN, and GK: conception and design and collection and assembly of data. EG, MB, BO, SB, UN, and GK: data analysis and interpretation. All authors: manuscript writing and final approval of manuscript.

## Conflict of Interest

The authors declare that the research was conducted in the absence of any commercial or financial relationships that could be construed as a potential conflict of interest.
