# Supplementary material for: Immunohistochemistry and Radiomic Features for Survival Prediction in Small Cell Lung Cancer
Source: Front Oncol. 2020 Aug 12;10:1161. doi: 10.3389/fonc.2020.01161 (PMC7438800; doi:10.3389/fonc.2020.01161)
Supplement: Supplementary file 1 [file Table_1.DOCX]

**Supplementary Table 1: Methodological information about the LDH and NSE measurements**

A. LDH

Assay:             Roche Lactate hydrogenase acc. to IFCC ver. 2

Analyser:        Roche cobas 8000, c702

Lactate dehydrogenase catalyzes the conversion of L‑lactate to pyruvate; NAD is reduced to NADH in the process. L‑Lactate+NAD --- LDHà Pyruvate+NADH+H+

The initial rate of the NADH formation is directly proportional to the catalytic LDH activity. It is determined by photometrically measuring the increase in absorbance.

B. NSE

Assay:             Roche Elecsys NSE

Analyser:        Roche cobas 8000, e801

Immunological in-vitro test for the quantitative determination of neuron-specific enolase (NSE) in human serum. Sandwich principle, ECLIA (ElectroChemiLuminescence-ImmunoAssay). The results are determined on the basis of a calibration curve.
