# Supplementary material for: Immunohistochemistry and Radiomic Features for Survival Prediction in Small Cell Lung Cancer
Source: Front Oncol. 2020 Aug 12;10:1161. doi: 10.3389/fonc.2020.01161 (PMC7438800; doi:10.3389/fonc.2020.01161)
Supplement: Supplementary file 2 [file Table_2.DOCX]

**Supplementary table 2: Summary of antibody protocols used.**

| **Antigen** | **Antibody** | **Antigen Retrieval** | **Antibody Concentration** | **Incubation time** |
| --- | --- | --- | --- | --- |
| **CD44** | clone 156-3C11, Cell Signalling | pH 6.1 | 1:500 | 30 minutes |
| **CD56** | clone 1B6, Dako | pH 9 | Prediluted | 20 minutes |
| **Chromogranin A** | clone DAK-A3, Dako | pH 9 | 1:200 | 30 minutes |
| **HIF1-a** | clone MAB5382, Chemicon | pH 6.1 | 1:500 | 60 minutes |
| **GLUT1** | clone SPM498, Abcam | pH 6.1 | Prediluted | 16 minutes |
| **KI-67** | clone MIB-1, Dako | pH 6.1 | Prediluted | 20 minutes |
| **PD-1** | Polyclonal goat, R&D Systems | pH 9 | 1:100 | 30 minutes |
| **PD-L1** | clone E3L1n, Cell Signalling | pH 9 | 1:200 | 30 minutes |
| **Synaptophysin** | clone DAK-Synap, Dako | pH 6.1 | Prediluted | 20 minutes |
| **TTF-1** | clone 8G7G3/1, Dako | pH 9 | Prediluted | 10 minutes |
